# Shear bond strengths of composite resin and giomer to mineral trioxide aggregate at different time intervals

**DOI:** 10.4317/jced.53791

**Published:** 2017-07-01

**Authors:** Amir-Ahmad Ajami, Mahmoud Bahari, Arezoo Hassanpour-Kashani, Mehdi Abed-Kahnamoui, Ayda Savadi-Oskoee, Farhad Azadi-Oskoee

**Affiliations:** 1 Assistant Professor, Department of Operative Dentistry, Faculty of Dentistry, Tabriz University of Medical Sciences, Tabriz, Iran; 2 Dental and Periodontal Research Center, Faculty of Dentistry, Tabriz University of Medical Sciences, Tabriz, Iran; 3 Endodontist, Private Practice, Tabriz, Iran; 4 Associate Professor, Department of Operative Dentistry, Faculty of Dentistry, Tabriz University of Medical Sciences, Tabriz, Iran; 5 General Dentist, Department of Operative Dentistry, Faculty of Dentistry, Tabriz University of Medical Sciences, Tabriz, Iran

## Abstract

**Background:**

The efficacy of the bond between the restorative materials and the pulp capping materials has an important role in the success of vital pulp therapy. Therefore, the aim of this study was to evaluate the shear bond strength of composite resin and giomer to MTA at different time intervals after mixing of MTA.

**Material and Methods:**

Ninety cylindrical MTA samples were prepared and assigned to two groups (n=45) based on the restorative materials used (composite resin or giomer). Each group was subdivided into 3 subgroups (n=15) based on the evaluation intervals (immediately, 2.45 hours and 3 days after mixing MTA). After the bonding procedures, the shear bond strengths of the samples were measured in MPa at a strain rate of 0.5 mm/min. Data were analyzed with repeated-measures ANOVA, post hoc tests and t-test (*P*<0.05).

**Results:**

Bond strength of composite resin was minimum at baseline but it increased significantly 2.45 hours after mixing MTA (*P*=0.002), with no significant changes in bond strength up to three days (*P*=0.08). Bond strength of giomer did not exhibit any significant changes from baseline to 2.45 hours after mixing MTA (P=078); however, at 3 days it reached a minimum (*P*=0.000). In addition, the means of bond strength of composite resin 2.45 hours and 3 days after mixing were significantly higher than those of giomer (*P*=0.001 and *P*=0.000, respectively).

**Conclusions:**

Bond strengths of composite resin 2.45 hours and also 3 days after mixing were significantly higher than those of giomer. In addition, the shear bond strength of giomer decreased over time; however, the shear bond strength of composite resin increased.

** Key words:**Composite resin, Giomer, Shear bond strength, Vital pulp therapy.

## Introduction

In recent years, vital pulp therapy has attracted special attention in dentistry, especially in endodontics (1). The aim of vital pulp therapy is to preserve the health and vitality of the dental pulp after traumatic injuries and exposure of the pulp due to caries (2). Historically, this treatment modality is carried out with the use of calcium hydroxide; however, due to its unpredictable results, it has not been widely accepted and adopted (3).

Introduction of new dental materials, which is supported by scientific evidence, has resulted in an increase in the cases of vital pulp therapy in recent years (1,4). In this context, mineral trioxide aggregate (MTA) has attracted special attention in vital pulp therapy, which is due to its proper biologic properties and favorable clinical and histological outcomes (5). MTA is superior to calcium hydroxide in relation to the induction of dentinogenesis in human dental pulp tissue (6). In addition, the pulp heals faster with the use of MTA compared to calcium hydroxide (7). In direct pulp cap (DPC) procedures, MTA has exhibited better efficacy compared to calcium hydroxide (8).

An important consideration is the fact that after a DPC procedure the restoration area should be completely sealed with an appropriate restorative material to prevent subsequent bacterial contamination (9,10). However, the majority of researchers who have suggested the use of MTA for vital pulp therapy purposes have recommended no restorative material and adhesive system as appropriate for permanent restoration of the cavity, nor have they suggested any time interval as appropriate for the final restoration after mixing and placing it in the pulp cap area (11).

In one of the few relevant studies, Tunch *et al.* showed that the etch-and-rinse adhesive systems exhibited higher shear bond strength between MTA and composite resins compared to the self-etch systems (12). On the contrary, Neelakantan *et al.* concluded that the bond strength of composite resin samples with a single-step self-etch bonding protocol immediately after mixing MTA was significantly higher than that achieved with etch-and-rinse and two-step self-etch systems at 45-minute and 24-hour intervals after mixing (11).

During the past decade, a new group of restorative materials, referred to as giomers, has been introduced. Giomers are similar to conventional methacrylate-based composite resins; however, they contain inorganic fillers, measuring 0.01 to 0.5 µm, of pre-reacted glass-ionomer instead of pure glass or quartz fillers (13,14). Giomers are used in a manner similar to conventional composite resins, with the application of an adhesive system. In addition, they exhibit favorable esthetic appearance, easy polishing, strength, and release and recharging of fluoride. The clinical success of giomer restorations has been shown in various studies (13,15). Considering a lack of sufficient data in relation to comparison of the bond strength of giomers and composite resins to MTA, the present study was designed to evaluate and compare the shear bond strength of composite resin and giomer to MTA at different time intervals after mixing MTA, i.e. immediately, 2 hours and 45 minutes, and 3 days after mixing.

## Material and Methods

Sample size calculation 

Based on a study by Neelakantan *et al.*, the sample size was calculated at 90 (45 in each group) by considering µ2=38.5, µ1=38.4, S1=1.3, S2=1.91, α=0.05, study power=80% and d=0.75 (11).

-Study Design

After the study protocol was approved by the Ethics Committee of the University, first 90 cylindrical acrylic resin samples, measuring 1 cm in diameter and 25 cm in height, were prepared. In order to create a completely smooth and horizontal surface, the cylinders were meticulously trimmed in a trimmer. Then cavities were created at the center of the cylinders, measuring 4 mm in diameter and 2 mm in height.

The MTA powder was mixed with distilled water based on manufacturer’s instructions in a Dappen dish to a thick creamy consistency. Then the paste was placed in the cavities in the acrylic resin molds using a carrier and packed with a condenser, followed by smoothening its surface with a spatula. To create identical surface roughness in all the MTA samples, the surfaces were polished with 600-grit abrasive paper before the bonding procedures.

Then the samples were randomly assigned to two groups based on the restorative material used: composite resin or giomer (n=45). Then the samples in each group were subdivided into 3 subgroups (n=15) based on the setting procedure duration: immediately, 2.45 hours and 3 days as follows:

Subgroup 1: Composite resin, immediately 

Subgroup 2: Composite resin, 2.45 hours 

Subgroup 3: Composite resin, 3 days

Subgroup 4: Giomer, immediately

Subgroup 5: Giomer, 2.45 hours

Subgroup 6: Giomer, 3 days

In MTA samples of subgroups 1 and 4, the bonding procedures were carried out immediately after placing MTA in the cavities of acrylic resin molds. All the other samples were placed vertically within a plastic container and 1 cm of water was poured in the container. After placing the lid of the container tightly, the samples in subgroups 2 and 5 and in subgroups 3 and 6 were incubated at 37°C for 2.45 hours and 3 days, respectively.

In subgroups 1, 2 and 3, in order to bond composite resin samples to MTA surfaces, the surfaces of MTA samples were etched with 35% phosphoric acid gel (Scotchbond Etchant, 3M ESPE, St Paul, MN, USA) for 15 seconds, rinsed with water for 30 seconds and then dried with oil- and moister-free air spray.

In the next stage, a clean microbrush (Microbrush Co, Greyton, WI, USA) was used to apply Adper Single Bond IITM one-bottle adhesive (3M ESPE, St Paul, MN, USA) to the prepared surfaces of the samples. The adhesive was applied in two layers based on manufacturers’ instructions and after application of each layer a mild air stream was applied on it for 2-5 seconds in order to vaporize the solvent. Then the adhesive layer was light-cured for 10 seconds using a Demetron A2 light-curing unit (KEEP CORPORAION 3225 Deming Way Suite 190 Middleton WI) at a light intensity of 1000 mW/cm2.

In subgroups 4, 5 and 6, in order to bond giomer to MTA surfaces, the self-etch Beautibond bonding agent (Shofu Inc., Kyoto 605-0983, Japan) provided by the giomer manufacturer was used. An adequate amount of the bonding agent was applied on the prepared MTA surfaces with the use of a clean microbrush. After 10 seconds, a 3-second mild air stream and then a strong current of air were applied to learn a third homogeneous adhesive layer. The Demetron A2 light-curing unit was used to light-cure the adhesive layer for 5 seconds at a light intensity of 1000 mW/cm2.

To achieve identical surfaces of composite resin and giomer in all the samples, transparent molds, measuring 3 mm in diameter and height, were used. To this end the transparent mold was placed on the surface of prepared MTA samples and in samples in group 1, Valux Plus composite resin (3M ESPE, St Paul, MN, USA) and in samples in group 2, giomer (Shofu Inc., Kyoto 605-0983, Japan) were placed within the mold with the use of a condenser, followed by light-curing for 20 seconds with the use of Demetron A2 light-curing unit at a light intensity of 1000 mW/cm2 from the occlusal aspect; finally, the whole mass was light-cured again for 40 seconds. Then the samples were incubated at 37°C and 100% relative humidity for 24 hours (11).

-Shear bond strength testing

In the final stage, the shear bond strength of the samples were determined in MPa in a universal testing machine (Hounsfield Test Equipment, Model H5KS, Surrey, UK) at a strain rate of 0.5 mm/min.

-Fracture pattern determinations

**Figure 1:**
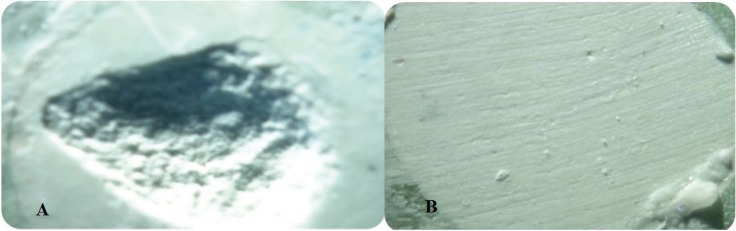
Failure modes of interfaces between MTA with composite resin and giomer; A: Cohesive, B: Adhesive.

Failure modes were determined under a stereomicroscope at ×10 and based on observations were divided into two adhesive or cohesive failure modes (Fig. 1).

-Statistical analysis of data

Data were analyzed with repeated-measures ANOVA, LSD tests and t-test, using SPSS 20. Statistical significance was set at *P*<0.05.

## Results

 Table 1 presents the means and standard deviations of shear bond strength values of composite resin and giomer to MTA at different time intervals and the failure modes in the study groups.

**Table 1:**
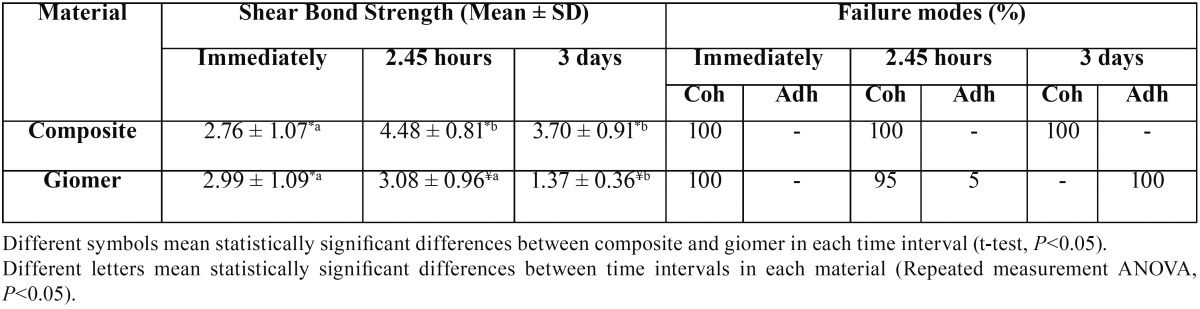
The means ± standard deviations and failure modes of shear bond strength tests.

Kolmogorov-Smirnov test showed normal distribution of data (*P*=0.93). In repeated measurements, considering the results of Mauchly’s sphericity test (the hypothesis of covariance homogeneity) (*P*=0.58), Greenhouse-Geisser test was used to evaluate the shear bond strength over time; the results showed that during the 3-day period the shear bond strength of composite resin (*P*=0.001) and giomer (*P*=0.000) to MTA changed significantly. Therefore, LSD tests were used for two-by-two comparisons of the time intervals in relation to shear bond strength values.

Evaluation of the shear bond strength of composite resin to MTA over time with LSD tests showed that.

-The shear bond strength immediately after mixing MTA was significantly less than that 2.45 hours (*P*=0.002) and 3 days (*P*=0.03) after mixing.

-The shear bond strengths 2.45 hours and 3 days after mixing were not significantly different (*P*=0.08) (Fig. 1).

In addition, evaluation of the bond strength of giomer to MTA over time with LSD tests showed that:

-The shear bond strength immediately and 2.45 hours after mixing were not significantly different (*P*=0.78).

-The shear bond strength 3 days after mixing was significantly less than that immediately (*P*=0.000) and 2.45 hours after mixing (*P*=0.000) (Fig. 1).

In addition, comparison of the shear bond strength of composite resin and MTA at each time interval with the use of t-test showed that:

-Immediately after mixing, there was no significant difference in shear bond strength between composite resin and giomer (*P*=0.57).

**Figure 2:**
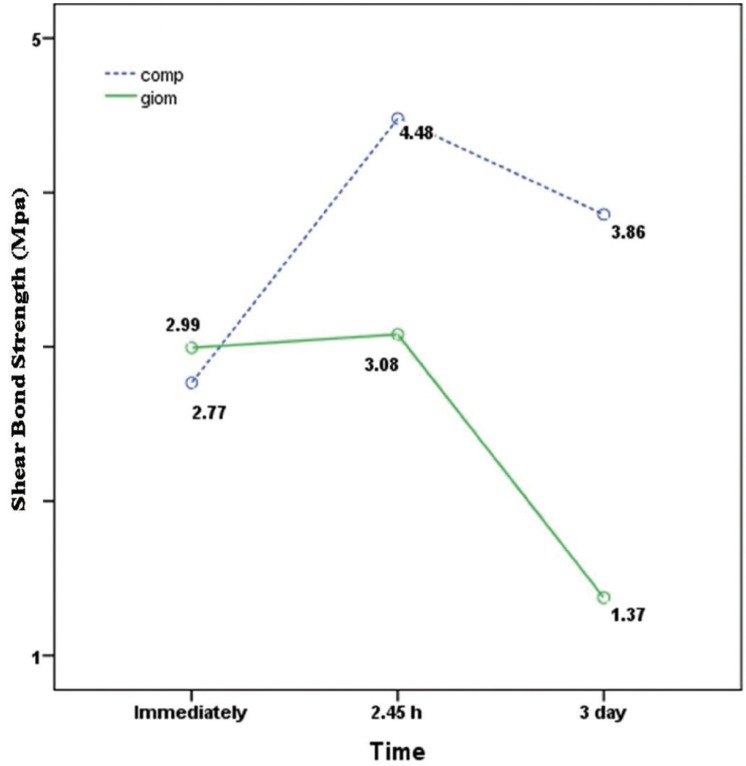
Comparison of the shear bond strengths of giomer and composite resin at different time intervals.

-At 2.45-hour and 3 days after mixing, the shear bond strength of composite resin was significantly higher than that of giomer (*P*=0.001) (Figs. 1,2).

## Discussion

Literature on the direct pulp capping procedures indicates that one of the most important factors in the prognosis of pulp-capped teeth is to place a microleakage-free restoration immediately after the procedure. Therefore, the bond strength between the restorative material and the cavity liner is one of the most important factors in the quality of tooth restorations and the response of the pulp to the pulp capping procedure (16). Absence of a proper bond with a hermetic seal results in microleakage and penetration of bacteria into the pulp, giving rise to the failure of vital pulp therapy (9,10).

In adhesive dentistry, microtensile shear bond strength (µTBS) test has been recommended for the evaluation of the bond strength. However, since MTA is a brittle material it is not possible to carry out µTBS on it. Therefore, in the present study shear bond strength test, which has been used in the majority of previous studies, was used (11).

Since the initial setting time of MTA is 2 hours and 45 minutes and its final setting time is approximately 3 days (17), one basic question for any clinician is whether or not it is possible to avoid a 2-session treatment, which is considered a risk factor for the success of vital pulp therapy, by placing composite resin or another adhesive material on the MTA immediately after mixing and placing it in the pulp capping area.

The results of the present study showed that the shear bond strength of composite resin (with E&R adhesive system) to MTA was low immediately after mixing; however, it significantly increased 2.45 hours after mixing. In addition, the bond strength to completely set MTA (3 days) was not significantly different from that after the initial setting (2.45 hours).

On the other hand, the bond strength of giomer (one-step self-etch adhesive system) immediately and 2 hours and 45 minutes after mixing was similar and higher than that 3 days after mixing.

SEM studies have shown that etching the surface of WMTA with phosphoric acid results in the erosion of crystalline structures and creation of a cracked surface with internal cracks (18,19). In contrast, use of self-etch systems does not result in the formation of cracks or pores on the MTA surface, although it destroys the crystalline structure. Therefore, it appears greater surface roughness is the reason for higher bond strength of composite resin to MTA with the use of ER adhesive systems compared to the use of two-step self-etch adhesive systems. Similar results have been reported in several previous studies (12,20).

The decrease in bond strength over time in the giomer group might be attributed to the inability of the self-etch adhesive system to penetrate into the completely set MTA because in this system no separate step exists for etching with phosphoric acid and its mechanism of action depends on simultaneous etching, priming and bonding. In contrast, in the present study, the bond strength of composite resin increased over time, which might be attributed to the greater stability of the interfacial layer resulting from the penetration of the adhesive into the set MTA compared to the interfacial layer, resulting from the penetration of the adhesive into MTA immediately after mixing.

Neelakantan *et al.* showed that the bond strength of composite resin to MTA with the use of E&R and one-step and two-step self-etch adhesive systems 24 hours after mixing decreased significantly compared to that immediately and 45 minutes after mixing (11). However, in the present study, the bond strengths of composite resin with the use of E&R adhesive system and giomer with the use of one-step self-etch adhesive system immediately after mixing were not different significantly. Nonetheless, at 2-hour-and-45-minute and 3-day intervals the bond strength of composite resin was significantly higher than that of giomer.

Oskoee *et al.* showed that the type of the adhesive system) E&R vs. 2-step self-etch) had no effect on the bond strength of composite resin to MTA after complete setting (10). In contrast, in a study by Shin *et al.*, the highest bond strength of composite resin to MTA was achieved with the use of Adhes E One F one-step self-etch adhesive system; however, there was no significant difference between the system above and 3-step and one-bottle E&R systems, with significantly higher bond strength compared to two-step self-etch adhesive systems (18). Neelakantan, too, reported a higher bond strength of composite resin to MTA with the use of one-step self-etch adhesive systems, which was attributed to factors such as pH or better wetting ability due to the simultaneous presence of solvents such as water and alcohol, resulting in a decrease in contact angle (11). The pH of the giomer system is 2.4. In addition, it lacks HEMA and acetone has replaced water and alcohol in its structure. Ethanol-based adhesive systems have higher bond strength compared to acetone-based adhesives (R and the internal angles). In addition, some of the properties of acetone, such as high volatility and rapid evaporation when the bottle’s cap is removed, make it an inappropriate solvent (21). In the self-etch adhesive systems, usually ethanol and acetone are used in combination with water as a solvent. A combination of ethanol or acetone with water is referred to as azeotropic, a property that forms hydrogen bonds between ethanol or acetone and water, facilitating the evaporation of this composition (22,23); however, the capacity of the formation of hydrogen bonds with the ketone (C=O) in acetone is less than that of ethanol (OH-) (24).

Another interesting finding of the present study was the fact that the bond strengths of giomer and composite resin to MTA immediately after mixing were not significantly different from each other, consistent with the similar fracture patterns of MTA in the two groups (100% cohesive). In contrast, at 2.45-hour and 3-day intervals, the bond strength of composite resin to giomer was significantly higher than that of giomer, as confirmed by fracture patterns because the higher number of adhesive failures indicates a weak bond strength. Adhesive failures in 2.45-hour and 3-day giomer groups increased in number, with the least resin bond strength in the 3-day giomer group between the study groups, and all the fractures were adhesive. In addition, the distribution pattern of fractures was highly consistent with the giomer bond strength; in this context, the 100% cohesive pattern in the immediate group converted to 100% adhesive fracture in the 3-day group.

Restorations subsequent to pulp capping procedures have different interfaces due to the multiplicity of the materials used, and the efficacy of the seal of each interface is important in the final success of the treatment. The bond strength values of different adhesive systems have been reported to be 13-35 MPa and the recommended bond strength values to achieve a restoration with no gaps with a proper seal has been reported to be 17‒20 MPa (25,26), which is much higher than bond strength values of giomer and composite resin with MTA in the present study. On the other hand, several studies have reported that the bond strength of conventional glass-ionomer (GIC) to MTA is high for two reasons: Firstly, the surface of MTA is rich in metallic oxides with which the GIC can form chemical bonds through strong metallic bonds; secondly, porosities on the MTA surface increase the surface area of micromechanical interlocking between MTA and GIC (27-29).

However, studies have shown this interesting finding that if GIC is bonded to the MTA surface after mixing MTA, it will undergo pre-test failures (11) and it has been recommended that if a decision is made to cover the surface of MTA with GIC, this should be done at least 45 minutes after mixing and placement of MTA in the pulp cap area (20). Oskoee *et al.* reported that when MTA are used as pulp capping agents, it is advisable to cover them with RMGI before restoration with composite resin because the bond strength of RMGI to composite resin is significantly higher than that to MTA and CEM (9).

The chief limitation of the present study was a lack of evaluation of the characteristics and surface morphology of WMTA at different time intervals, which might have helped explain the results. Therefore, it is suggested that in future studies SEM and AFM evaluation be carried out in order to further elucidate the reasons for decreases and increases in bond strength at different time intervals.
